# (-)-Carveol Prevents Gastric Ulcers via Cytoprotective, Antioxidant, Antisecretory and Immunoregulatory Mechanisms in Animal Models

**DOI:** 10.3389/fphar.2021.736829

**Published:** 2021-08-23

**Authors:** Catarina Alves de Lima Serafim, Maria Elaine Cristina Araruna, Edvaldo Balbino Alves Júnior, Leiliane Macena Oliveira Silva, Alessa Oliveira Silva, Marcelo Sobral da Silva, Adriano Francisco Alves, Aurigena Antunes Araújo, Leônia Maria Batista

**Affiliations:** ^1^ Postgraduate Program in Natural and Synthetic Bioactive Products, Health Sciences Center, Federal University of Paraíba (UFPB), João Pessoa, Brazil; ^2^ Department of Physiology and Pathology, Health Sciences Center, Federal University of Paraíba (UFPB), João Pessoa, Brazil; ^3^ Department of Morphology, Histology and Basic Pathology, Biosciences Center, Federal University of Rio Grande do Norte, Natal, Brazil

**Keywords:** (-)-Carveol, gastric ulcer, gastroprotection, cytoprotection, antioxidant, immunoregulatory mechanism

## Abstract

**Background:** (-)-Carveol (*p*-Mentha-6,8-dien-2-ol) is a monocyclic monoterpenic alcohol, present in essential oils of plant species such as *Cymbopogon giganteus*, *Illicium pachyphyllum* and in spices such as *Carum carvi* (cumin). Pharmacological studies report its antitumor, antimicrobial, neuroprotective, vasorelaxant, antioxidant and anti-inflammatory activity.

**Hypothesis/Purpose:** The objective of this study was to evaluate the acute non-clinical oral toxicity, gastroprotective activity of monoterpene (-)-Carveol in animal models and the related mechanisms of action.

**Methods:** Acute toxicity was assessed according to OECD guide 423 in mice. Ethanol, stress, NSAIDs and pylorus ligation-induced gastric ulcer models were used to investigate antiulcer properties. The related mechanisms of action were using the ethanol-gastric lesions protocol.

**Results:** (-)-Carveol has low toxicity, with a lethal dose 50% (LD_50_) equal to or greater than 2,500 mg/kg according to OECD guide nº 423. In all gastric ulcer induction methods evaluated, (-)-Carveol (25, 50, 100 and 200 mg/kg, p.o.) significantly reduced the ulcerative lesion in comparison with the respective control groups. To investigate the mechanisms involved in the gastroprotective activity, the antisecretory or neutralizing of gastric secretion, cytoprotective, antioxidant and immunoregulatory effects were evaluated. In the experimental protocol of pylorus ligation-induced gastric ulcer, (-)-Carveol (100 mg/kg) reduced (*p* < 0.001) the volume of gastric secretion in both routes (oral and intraduodenal). The previous administration of blockers NEM (sulfhydryl groups blocker), L-NAME (nitric oxide synthesis inhibitor), glibenclamide (K_ATP_ channel blocker) and indomethacin (cyclo-oxygenase inhibitor), significantly reduced the gastroprotection exercised by (-)-Carveol, suggesting the participation of these pathways in its gastroprotective activity. In addition, treatment with (-)-Carveol (100 mg/kg) increased (*p* < 0.001) mucus adhered to the gastric wall. Treatment also increased (*p* < 0.001) levels of reduced glutathione (GSH), superoxide dismutase (SOD) and interleukin-10 (IL-10). It also reduced (*p* < 0.001) malondialdehyde (MDA), myeloperoxidase (MPO), interleukin-1 beta (IL-1β) and tumor necrosis factor-alpha (TNF-α) levels.

**Conclusion:** Thus, it is possible to infer that (-)-Carveol presents gastroprotective activity related to antisecretory, cytoprotective, antioxidant and immunomodulatory mechanisms.

## Introduction

Peptic ulcer is a common disease of global incidence and prevalence, affecting millions of people worldwide and showing high rates of recurrence (Lanas and Chan, 2017). This disease is of complex and multifactorial origin and develops from the loss of balance between aggressive (hydrochloric acid, continuous use of non-steroidal anti-inflammatory drugs-NSAIDs, smoking, oxidative stress and the abusive ethanol intake) and protective agents (mucus, bicarbonate, prostaglandins, nitric oxide, growth factors and cell renewal) of the mucosa of the gastrointestinal tract (Yandrapu and Sarosiek, 2015; Malfertheiner and Schulz, 2020).

Among the therapeutic options available for this condition are antacids, cytoprotectors, H2 histamine receptor antagonists, proton pump inhibitors (PPIs) and in cases where there is infection by *Helicobacter pylori*, it is necessary to use antimicrobial drugs (Sałaga and Mosińska, 2017). However, these medications are associated with a variety of side and adverse effects and poor healing of ulcers and, in turn, the recurrence of ulcers, generating a high economic impact for users and public health systems (Byrne et al., 2018; Kavitt et al., 2019).

Natural products, including plants, animals, microorganisms and minerals, have historically proven their value as a source of molecules with therapeutic potential (Rao et al., 2019). As well, many natural products have served as inspiration and starting points for medicinal chemistry and the development of new molecules and drugs (Barreiro, 2019).

The scientific investigation of natural products, especially those obtained from plant species, has increasingly attracted the attention of researchers and the pharmaceutical industry, mainly due to the great chemical and structural diversity of their phytochemicals and the variety of biological effects, exerting an impact remarkable in the development of medicines (Prieto-Martínez et al., 2019).

Currently, approximately 35% of the medicines launched in the pharmaceutical market come from natural products. Furthermore, most of the molecules involved in clinical trials are also of natural origin (Newman and Cragg, 2016). The global market for these drugs is estimated to be around $ 1.1 trillion (Calixto, 2019).

Several medicinal plant extracts and their isolated constituents such as alkaloids, flavonoids, phenylpropanoids and terpenes (Dutra et al., 2016) have already evidenced many biological activities and have been explored in the prevention and treatment of the most diverse human affections (Tetali, 2019).

Terpenes, also called terpenoids or isoprenoids, comprise the class of secondary metabolites with the greatest chemical diversity (Christianson, 2017). The biological activities of the several classes of terpenes (monoterpenes, sesqui-, di-, tri- and tetraterpenes and their glycosides) have been evidenced *in vitro* and *in vivo* studies (Wojtunik-Kulesza et al., 2019).

(-)-Carveol, chemically known as p-Mentha-6,8-dien-2-ol, is a natural unsaturated monocyclic monoterpenic alcohol, found as a constituent of essential oils of many plants, such as *Cymbopogon giganteus* (Bossou et al., 2013), *Illicium pachyphyllum* (Liu et al., 2012) and *Carum carvi* (cumin) (Fang et al., 2010). This phytoconstituent showed some pharmacological activities such as antitumor (Wagner and Elmadfa, 2003), antibacterial, antifungal (Guimarães et al., 2019), neuroprotective (Hritcu et al., 2020), antioxidant and anti-inflammatory (Kaur et al., 2019; Marques et al., 2019).

Thus, considering the previous promising results, this study aimed to evaluate the oral acute toxicity, gastroprotective activity and the mechanisms of action of (-)-Carveol using different animal models of acute induced gastric ulcers.

## Materials and Methods

### Animals

Male Swiss mice (*Mus musculus*) (25–35 g) and male Wistar rats (*Rattus norvegicus*) (180–250 g) were provided by the Animal Production Unit (APU) of the Institute for Research on Drugs and Medicines of Federal University of Paraiba (IPeFarM/UFPB). The animals were acclimated to the conditions of the local bioterium in a temperature of 23 ± 2°C and a light-dark cycle of 12 h, fed with industrial pellet food and water ad libium. All experimental protocols followed international principles for the study with laboratory animals (Zimmermann, 1983) and were approved by the Institutional Ethics Commission on Animal Use (CEUA/UFPB) under registration number: 4881190619. All efforts were to reduce the number of animals used, their pain, suffering and stress.

### Reagents

The following substances were used in this study: (-)-Carveol (SIGMA Chemical Co., United States), sodium acetate (SIGMA Chemical Co., United States), acetonitrile (SIGMA Chemical Co., United States), [4-(2-hydroxyethyl)-1-piperazineethanesulfonic acid] (HEPES) (SIGMA Chemical Co., United States), 5,5′-ditiobis-2-nitrobenzoic acid (DTNB) (SIGMA Chemical Co., United States), hydro-chloric acid (MERCK, Germany), ethylenediaminetetraacetic acid (EDTA) (SIGMA Chemical Co., United States), bovine serum albumin (BSA) (SIGMA Chemical Co., United States), alcian blue (SIGMA Chemical Co., United States), glacial acetic acid (SIGMA Chemical Co., United States), trichloroacetic acid (SIGMA Chemical Co., United States), anti-rat antibodies for IL-1β, TNF-α and IL-10 (R&D systems), biotinylated sheep polyclonal antibodies (anti-IL-1β, anti-TNF-α or an-ti-IL-10) (R&D Systems) carbenoxolone (SIGMA Chemical Co., United States), sodium carbonate (MERK, Germany), potassium chloride (SIGMA Chemical Co., United States), magnesium chloride (SIGMA Chemical Co., United States), sodium chloride PA (QUIMEX-MERCK, Brazil), ethanol (MERCK, Germany), ethyl ether (MERCK, Germany), phenolphthalein (RIEDELDE HAËN, Germany), monobasic sodium phosphate (SIGMA Chemical Co., United States), bibasic sodium phosphate (SIGMA Chemical Co., United States), fluoride sodium (SIGMA Chemical Co., United States), glibenclamide (SIGMA Chemical Co., United States), sodium hydroxide (QUIMEX-MERCK, Brazil), indomethacin (SIGMA Chemical Co., United States), lansoprazole (SIGMA Chemical Co., United States), Methyl-Phenylindole (SIGMA Chemical Co., United States), Misoprostol (ALVAC®), methylmaleimide (SIGMA Chemical Co., United States), N-nitro-l-arginine-methyl-ester (SIGMA Chemical Co., United States), omeprazole (SIGMA Chemical Co., United States), piroxicam 20 mg (HEXAL, Brazil), ranitidine (SIGMA Chemical Co., United States), ketamine 5% (VETANARCOL), sucrose (SIGMA Chemical Co., United States), Tris Buffer (Vetec®), Trizma Buffer (SIGMA Chemical Co., United States) and xylazine 2% (DORCIPEC).

### Evaluation of the Acute Oral Toxicity

(-)-Carveol was submitted to the acute toxicity protocol according to OECD (OECD, 2002) and Almeida et al. (1999). Mice (*n* = 3 per group) were fasted for 4 h and treated (p.o.) with 5% tween 80 (10 ml/kg-control group), (-)-Carveol (300 mg/kg or 2000 mg/kg), and then once a day for 14 days of experimentation. After treatment, behavioral effects were observed in the first 4 h. Parameters such as feed consumption, water intake and body weight were also evaluated during 14 days after administration of (-)-Carveol. By the end, the animals were euthanized and the main organs (heart, liver, spleen and kidneys) were removed to calculate the organ index, using the organ weight in milligrams (mg) by the animal’s weight in grams (g). The death number of the animals during the trial period was used to estimate the Lethal dose 50% (LD50) as recommended by OECD guide 423 (OECD, 2002).

### Evaluation of Gastroprotective Activity

#### Ethanol-Induced Gastric Ulcer

This experimental model was carried out as recommended by Morimoto et al. (1991). Rats (*n* = 7 per group) were fasted for 24 h and distributed in four groups: normal (no treatment), control group (5% tween 80–10 ml/kg), carbenoxolone (100 mg/kg-standard cytoprotective drug) and (-)-Carveol (25, 50, 100 and 200 mg/kg). After 1 h of an oral pre-treatment, the ulcerative lesion was induced by the administration of absolute ethanol (4 ml/kg-p.o.). After 60 min, the animals were euthanized, their stomachs opened along the great curvature and photographed (12-megapixel dimension) for ulcerative lesions quantification as ulcerative lesion area (ULA) the software AVSoft Bioview Spectra 4.0® (AvSoft, Brazil) were used. Furthermore, fragments of gastric tissue from each group were collected, immediately immersed in liquid nitrogen and stored at −80°C for subsequent dosages of antioxidants and cytokines. Another portion of gastric tissue was removed and used in histomorphological studies.

##### Histological Analysis

Fragments of gastric tissue from the ethanol-induced gastric ulcer protocol were preserved in a 10% buffered formaldehyde solution until histological processing. The tissues were embedded in histological paraffin and sectioned with a thickness of 4 µm. From the sectioned tissues, four stains were performed: hematoxylin and eosin (HE), toluidine blue, Masson’s trichrome and Periodic Acid-Schiff (PAS).

##### Morphometric Analysis

The histological sections stained by Masson’s trichrome and PAS were visualized through the ×40 objective of the Olympus microscope (Tokyo, Japan) and 20 random images were digitalized through the same microscope and Q-Color3 microscope, making a total area of 281,872 μm^2^ of gastric mucosa analyzed in each type of histochemical reaction. The area of extracellular matrix or mucins was calculated using algorithms built in the KS300 software contained in the Carl Zeiss image analyzer (Oberkochen, Germany). In each image, all pixels with shades of blue (Masson’s trichrome) or bonina (PAS) were selected to create a binary image, digitally processed and calculate the area in μm^2^. To calculate the number of mast cells, a histological section of the stomach stained by the toluidine blue of each case was visualized through the ×40 objective for the digitization of 20 random areas through the micro camera. Using the same images, all cells were counted interactively using the same program (Caliari, 1997).

#### Stress-Induced Gastric Ulcer (Immobilization and Cold)

This model was carried out according to the methodology described by Levine (1971). Mice (*n* = 7 per group) were fasted for 24 h and pretreated (p.o.) with 5% tween 80 solution (10 ml/kg-group control), ranitidine (50 mg/kg-standard drug) and (-)-Carveol (25, 50, 100 and 200 mg/kg). After 30 min, the animals were immobilized by the front and rear legs and placed in PVC containers (9 cm long × 3.5 cm in diameter) and kept 3 h in a temperature of 4 ± 1°C for inducing gastric ulcers. After that the animals were euthanized, their stomachs removed and opened to determine the ulcerative lesion index (ULI).

ULI = ∑ (score 1 × 1) + (score 2 × 2) + (score 3 × 3)

Score 1: hemorrhagic stitches and ulcerations up to 1 mm.

Score 2: ulcerations with 2 mm.

Score 3: deep ulcerations of 3 mm or more.

#### NSAID-Induced Gastric Ulcer

This experimental protocol was conducted according to the model proposed by Puscas (Puscas et al., 1997). For this, mice (*n* = 7 per group) subjected to 24 h fast were pretreated (p.o.) with 5% tween 80 solution (10 ml/kg-group control), ranitidine (50 mg/kg-standard drug) and (-)-Carveol (25, 50, 100 and 200 mg/kg). After 30 min, the gastric ulcer was induced by the administration of piroxicam (30 mg/kg, s.c.). After 4 h the mice were euthanized, the stomachs removed and opened to determine the ULI.

#### Pyloric Ligation-Induced Gastric Ulcer and Gastric Secretion Studies (Oral or Intraduodenal Route)

After 24 h fast, for the oral administration model, the rats (*n* = 10 per group) were pretreated (p.o.) with 5% tween 80 solution (10 ml/kg-group control), ranitidine (50 mg/kg-standard drug) and (-)-Carveol (100 mg/kg). After 30 min, the rats were anesthetized with ketamine hydrochloride (70 mg/kg-i.v.) and 2% xylazine hydrochloride (10 mg/kg-i.v.) and subjected to a longitudinal incision below the xiphoid process for ligation of the pylorus (gastric juice contention). Then, the stomachs were internalized and the abdominal cavity was sutured. In the intraduodenal administration model (i.d.), the same treatments described above were performed after the surgical ligation procedure. After 4 h of pyloric ligation, the rats were euthanized, their stomachs removed and opened to determine the ULI. This model was described by Shay (1945).

### Gastroprotective Mechanism

#### Anti-Secretory or Neutralizing Mechanism

##### Evaluation of Biochemical Parameters of Gastric Juice After Pyloric Ligation (p.o./I.d)

This protocol was based on the methodology described by Shay (1945), as previously described. After 4 h of pyloric ligation, the animals were euthanized, stomachs were opened, gastric content was collected and the following biochemical parameters were determined: potential of hydrogen (pH), H^+^ concentration ions (mEq/mL/4 h), and volume (ml). The pH was checked on a digital pH meter PG 2000 (GEHAKA, Brazil).

#### Cytoprotective Mechanisms

##### Involvement of Sulfhydryl Groups, Nitric Oxide, ATP-Dependent Potassium Channels and Prostaglandins

To investigate the cytoprotective mechanisms involved in the gastroprotective activity of (-)-Carveol the role of sulfhydryl groups (Matsuda et al., 1999), nitric oxide (Sikirić et al., 1997), KATP (de Olinda et al., 2008) and prostaglandins (de Araújo Rodrigues et al., 2010) was assessed. After 24 h fasting, rats were divided into six groups (*n* = 7 per group). Three groups received intraperitoneally 0.9% NaCl solution (10 ml/kg) the other three received 10 mg/kg N-ethylmaleimide (NEM-SH inhibitor), 70 mg/kg N-nitro-L-arginine methyl ester (L-NAME-nitric oxide synthase inhibitor), 5 mg/kg glibenclamide (KATP channel blocker) or 30 mg/kg indomethacin (cyclooxygenase blocker) by the same route. After 30 min, rats were treated orally with 5% tween 80 (control group), standard control drugs or (-)-Carveol (100 mg/kg). The standard drugs used to evaluate the participation of SHs and NO was carbenoxolone (100 mg/kg, orally), while 3 mg/kg diazoxide, a KATP activator, was employed intraperitoneally. To determine the role of prostaglandins, 50 μg/kg misoprostol, a prostaglandin analog was given orally. After 1 h, was administered orally absolute ethanol (4 ml/kg) to induce gastric lesions. After 60 min after induction, the animals were sacrificed, the stomachs were opened and photographed for ALU determination.

##### Determination of the Concentration of Mucus Adhered to the Gastric Wall

This model was conducted in accordance with Rafatullah et al. (1990) with modifications. After 24 h fast, rats (*n* = 7 per group) were divided into three groups and treated (p.o.) with 5% tween 80 solution (10 ml/kg), carbenoxolone (200 mg/kg) and the most effective dose of (-)-Carveol (100 mg/kg). After 1 h, the rats were anesthetized (5% ketamine hydrochloride and 2% xylazine) and submitted to a surgical procedure below the xiphoid apophysis so that the pylorus ligation. After 4 h, the animals were euthanized, the stomach removed and opened along the great curvature. The glandular portion of the stomach was separated, weighed and immersed for 2 h in 0.1% of alcian blue solution (dye that complexing with mucus). The excess of alcian blue was removed by washing the stomach (2x) with sucrose solution (0.25 mol/L), the first for 15 min and the second for 45 min. Complexed mucus dye adhered to the stomach wall was extracted with magnesium chloride (0.5 mol/L), stirring intermittently for 1 min, every 30 min, for 2 h. An aliquot of this solution was removed (approximately 3 ml) and an equal volume of ethyl ether (P.A.) was added, mixed with this solution, centrifuged at 3,600 rpm for 10 min and the supernatant discarded together with the mucus ring. From the remaining aqueous phase, absorbance was determined at 598 nm (MULTISCA model, BRAND LABSYSTEMS®). The determination of the concentration of alcian blue in the samples was made by interpolation in a standard curve with several known concentrations of alcian blue. The results were expressed in μg of alcian blue/g of tissue.

#### Antioxidant’s Activity

##### Non-Protein Sulfhydryl Group Determination

To determine the GSH levels, the protocol according to Faure and Lafond (1995) was followed. The tissue samples were suspended in 0.02 M 1:10 (v/v) EDTA. From this homogenate, 400 µl were removed and 320 µl of distilled water and 80 µl of 50% trichloroacetic acid were added, being centrifuged at 3,000 rpm at 4°C for 15 min. Then, 100 µl of the resulting supernatant was pipetted into a 96-well microplate and 200 µl of Tris and 25 µl of DTNB were added. This microplate was incubated at room temperature and after 15 min, a reading on a spectrophotometer (Polaris) was performed at a wavelength of 412 nm. The calibration curve was made with reduced L-glutathione. The GSH values of the samples were calculated by interpolating the values with the standard curve and expressed in nmol GSH/mg of protein.

##### Malondialdehyde Determination

The samples were suspended in Trizma® buffer (Tris HCl) 1:5 (w/v), homogenized and centrifuged at 11.000 rpm at 4°C for 10 min. Soon after, 300 μl of the supernatants were transferred to Eppendorf tubes and 750 μl of the chromogenic compound (10.3 mM of 1-methyl-2-phenylindol) and 225 μl of hydrochloric acid (37%) were added and incubated at 45°C in a water bath for 40 min and again centrifuged at 11.000 rpm at 4°C for 5 min. Then, 300 μl of the supernatant was transferred to a 96-well microplate and absorbance was determined by colorimetry (586 nm), using a plate reader (Polaris’ spectrophotometer). The data were interpolated with the standard curve and the results were expressed as nmol MDA/g tissue (Esterbauer and Cheeseman, 1990).

##### Myeloperoxidase Determination

The tissue fragments were homogenized in the hexadecyltrimethylammonium bromide (HTAB) buffer, which has a detergent function, lysing the granules of the neutrophils that contain the myeloperoxidase. After homogenization, the material was subjected to sonication for 5 min. Then, the sample was subjected to a double freezing and thawing process to facilitate the disruption of cellular structures and consequently the release of the enzyme. The homogenate was centrifuged at 5.000 rpm at 4°C for 20 min and concentrated for 24 h. On the following day, 7 μl of the supernatant were collected, to which 200 μl of the reading solution (o-dianisidine hydrochloride, potassium phosphate buffer and 1% H_2_O_2_) were added. The reading was performed using a spectrophotometer at 450 nm wave, at times 0 and 1 min. The results were expressed as units of myeloperoxidase per gram of tissue (Krawisz et al., 1984).

##### Superoxide Dismutase Determination

The tissue samples were homogenized in phosphate buffer (0.4 M, pH 7.0) and centrifuged for 15 min at 10.000 rpm at 4°C. The supernatant was removed and used in the assay. The plates containing the reaction medium (10 mM phosphate buffer), L-methionine (1.79 mg/ml, pH 7.8), riboflavin (0.2 mg/ml, pH 7.8), NBT (1.5 mg/ml, pH 7.8) and 10 μl of the sample supernatant were exposed to a fluorescent lamp (15 W) for 10 min. After this period, the material was taken to the 630 nm spectrophotometer (Sun et al., 1988).

#### Immunoregulatory Activity

##### Cytokines Determination

The levels of pro-inflammatory (IL-1β and TNF-α) and anti-inflammatory (IL-10) cytokines were determined by means of an ELISA immunoenzymatic assay (sandwich type). The capture antibodies for each interleukin were sensitized in a 96-well microplate (flat bottom). After 18 h, the plate was washed with 0.05% tween 20 solution (wash buffer), blocked with a 1% bovine serum albumin solution and washed with the wash buffer. Soon after, a tissue macerate was prepared in phosphate buffered saline (PBS) in the pro-portion of 100 mg of tissue to 600 μl of PBS, homogenized and centrifuged at 4,000 rpm for 10 min at 4°C. Supernatants (100 μl) were pipetted in a 96-well plate and made the standard curve. The biotinylated secondary antibody (100 μl) was added to each well, followed by incubation for 2 h and three washes. The plate was incubated with streptavidin for 20 min, washed 3 times, the substrate for development was added (DuoSet Kit^©^-R & D Systems Catalog-DY999) and incubated for 20 min. After this time, the reaction was stopped by adding 50 μl of the stop solution and the reading was performed on a spectrophotometer at 450 nm. The results were obtained by interpolation with the standard curve and expressed in pg/ml (Kendall et al., 1983).

### Statistical Analysis

The results were expressed as mean ± standard deviation (d.p.). To analyze the data obtained in the acute toxicity tests, the unpaired Student’s “t” test was performed. For the other experimental protocols, one-way analysis of variance (ANOVA) was used, followed by Dunnett and Tukey’s post-tests. The data were analyzed using the GraphPad Prism version 6.0 (San Diego, CA, United States) and the minimum significance level was *p*<0.05.

## Results

### Evaluation of Acute Toxicity and Median Ld50

The animals submitted to the administration of (-)-Carveol at doses of 300 and 2000 mg/kg (p.o.) did not show any behavioral changes (*p* > 0.0.5), in comparison to their respective control group (vehicle-5% tween 80 solution). There was no death in the group of animals treated with (-)-Carveol at doses of 300 and 2.000 mg/kg. With this, the LD50 of (-)-Carveol can be estimated at ≥2,500 mg/kg according to the OECD guide 423, being classified in category five according to the Global Harmonized Classification System (GHS). In addition, regarding weight evolution, organ index, water consumption and feed, there were also no significant changes (*p* > 0.0.5) in the groups of animals treated with (-)-Carveol (300 and 2000 mg/kg) compared to the control (5% tween 80) (Table 1).

**TABLE 1:** Effect of oral administration of (-)-Carveol on weight evolution, organ index, water and feed intake in male mice.

Parameters	Treatments		
Weight evolution (g)	5% Tween 80	(-)-Carveol 300 mg/kg	(-)-Carveol 2,000 mg/kg
Initial	26.33 ± 1.75	27.77 ± 1.41	26.83 ± 1.94
Final	30.83 ± 1.16	32.04 ± 1.41	31.83 ± 2.85
Organ index			
Liver	55.04 ± 3.25	56.16 ± 2.22	56.82 ± 2.53
Heart	5.19 ± 1.05	5.45 ± 1.06	5.24 ± 0.80
Kidneys	15.20 ± 2.01	14.37 ± 1.89	14.57 ± 2.10
Spleen	4.74 ± 0.91	4.92 ± 1.38	5.51 ± 1.76
Water consumption (ml)			
	25.39 ± 2.07	26.57 ± 1.45	26.18 ± 2.70
Feed consumption (g)			
	22.96 ± 1.97	23.14 ± 1.95	23.14 ± 2.41

The results are expressed as mean ± SD. (*n* = 6). Unpaired Student’s “t” test compared to the control group (5% tween 80). Weight was assessed on the 1st and 14th days. For organ evaluation, the values were expressed as an organ index that corresponds to the division of organ weight (mg) by animal weight (g).

### Ethanol-Induced Gastric Ulcer

**FIGURE 1:**
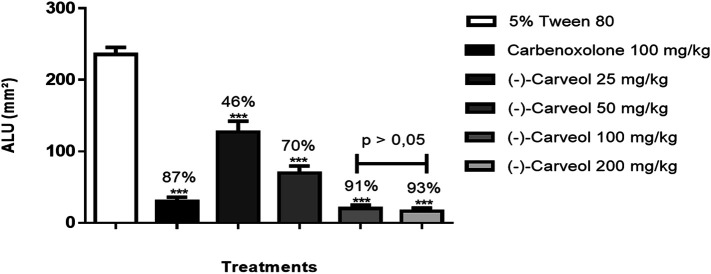
Effect of oral administration of carbenoxolone and (-)-Carveol in gastric ulcers induced by ethanol in rats. Results are expressed as mean ± SD. One-way analysis of variance (ANOVA); followed by Dunnett and Tukey's test was performed using the software Graph Pad Prism. ***p < 0.001 compared to the control group (5% tween 80) (n = 6−7). The bars above the 100 and 200 mg/kg doses indicate the existence or not of statistical differences in the Tukey test. ULA = Ulcerative Lesion Area.

**FIGURE 2:**
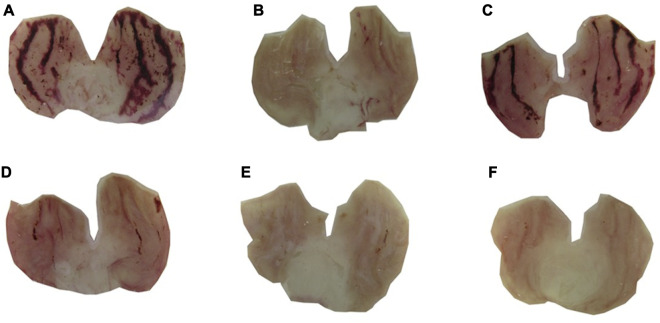
Macroscopic aspects of the gastric mucosa of male Wistar rats pretreated (p.o.) with 5% tween 80 **(A)**; carbenoxolone 100 mg/kg **(B)**; (-)-Carveol 25 mg/kg **(C)**, 50 mg/kg **(D)**, 100 mg/kg **(E)** and 200 mg/kg **(F)** in the ethanol induced gastric ulcer model.

In acute ethanol-induced gastric ulcer model, carbenoxolone (100 mg/kg) and (-)-Carveol (25, 50, 100 and 200 mg/kg, p.o.) significantly reduced ULA to 87% (30.0 ± 6.0 mm^2^), 46% (126.9 ± 15.3 mm^2^), 70% (69.9 ± 9.5 mm^2^), 91% (20.2 ± 4.7 mm^2^) and 93% (16.4 ± 4.5 mm^2^) respectively, when compared to the control group (235.7 ± 9.9 mm^2^) (Figures 1, 2). (-)-Carveol significantly reduced ULA in all doses tested, but the dose of 100 mg/kg was selected as the most effective, since it did not differ statistically from the dose of 200 mg/kg, but it differed from the other doses when evaluated by the Tukey test of multiple comparisons.

#### Histological Analysis

**FIGURE 3:**
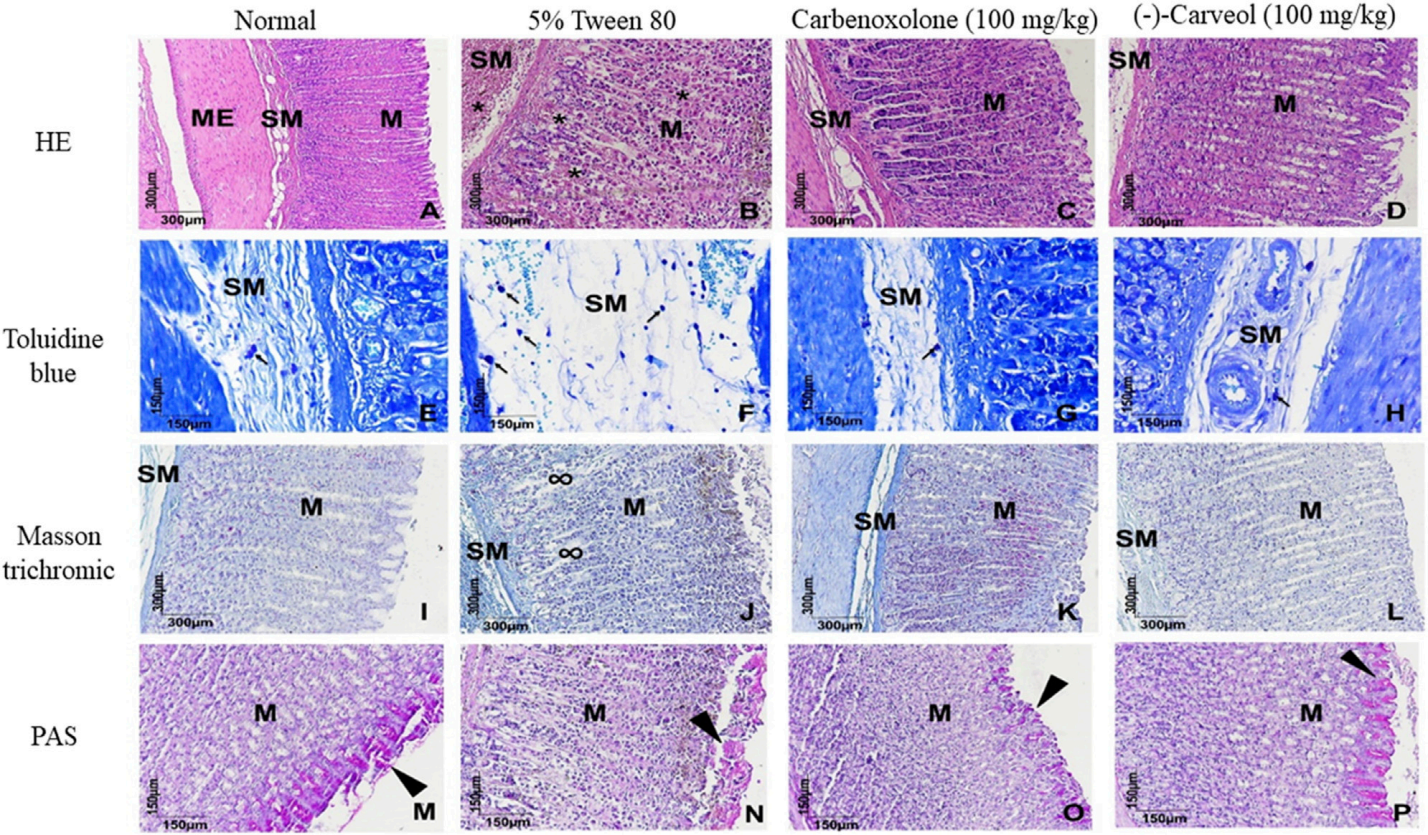
Microscopic effects of the gastric mucosa of rats submitted to gastric lesions induced by ethanol and pretreated or not with (-)-Carveol. Representative photomicrographs of the animals' stomachs from the experimental groups: Normal **(A,E,I,M)**, 5% tween 80 **(B,F,J,N)**, carbenoxolone 100 mg/kg **(C,G,K,O)** and (-)-Carveol 100 mg/kg **(D,H,L,P)**. HE staining **(A,B,C,D)**, toluidine blue **(E,F,G,H)**, Masson trichrome **(I,J,K,L)** and PAS **(M,N,O,P)**. Mucous layer (M), submucosal layer (SM), external muscle (ME), hemorrhage (*), mast cells (black arrows), extracellular matrix (∞) and mucins (arrowhead).

The stomach fragments stained in hematoxylin and eosin (HE) belonging to the normal, carbenoxolone and (-)-Carveol groups (Figures 3A,C,D), showed complete mucosa (M), characterized by the presence of cells parietal and main cells without cytostructural changes compatible with healthy tissue, which also extended to the submucosa (SM) and external muscle (ME). Different from the control group (5% tween 80) (Figure 3B), which presents a mucosa with dead parietal cells in a “loose” aspect of the other glandular components which are surrounded by hemorrhage (*), a lesion that extends to the submucosa. In the toluidine blue staining, it is observed that in the group pretreated with (-)-Carveol (Figure 3H), the mast cells are discretely distributed in regions close to the blood vessels (black arrows), different from what occurs in the control group (5% tween 80) (Figure 3F), where these cells are numerous and distributed through the swollen submucosa. In Mas-son’s trichrome staining, it was found that (-)-Carveol (Figure 3L) reduced the deposition of the extracellular matrix compared to the control group (5% tween 80) (Figure 3J). And by PAS staining it was observed that in the group (-)-Carveol (Figure 3P) the basic mucins are regularly distributed on the surface of the gastric glands, giving in some cases the aspect of acini in these regions. In the control group (5% tween 80) (Figure 3N), an irregular and more discreet distribution of these proteins was observed.

#### Effect of (-)-Carveol on the Number of Mast Cells, Extracellular Matrix and Mucins on the Gastric Mucosa of Rats Submitted to Gastric Lesions Induced by Ethanol

**FIGURE 4:**
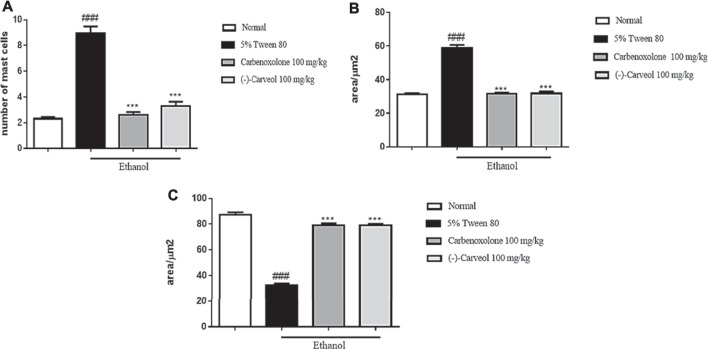
Effect of oral administration of carbenoxolone and (-)-Carveol on the number of mast cells **(A)**, extracelular matrix **(B)** and mucins **(C)** on the gastric mucosa of rats submitted to gastric ulcer model induced by ethanol. The values represent the mean ± SD. For statistical analysis, the one-way ANOVA test was used followed by the Tukey post test, where ***p < 0.001 vs. negative control; ###p < 0.001 vs. normal.

The control group (5% tween 80) showed a significant increase in the number of mast cells in the gastric mucosa to 8.9 ± 3.4 in relation to the normal group (2.3 ± 0.6). In contrast, carbenoxolone (100 mg/kg) and (-)-Carveol (100 mg/kg) showed a reduction in the number of mast cells to 2.6 ± 0.9 and 3.3 ± 1.0, respectively, compared to the control group (Figure 4A). Regarding extracellular matrix proteins, it was observed that in the control group (5% tween 80) there was a significant increase in these proteins to 58.9 ± 7.4 µm^2^ when compared to the normal group (31.2 ± 3.2 µm^2^). However, the amount of these proteins was significantly reduced with the previous administration of carbenoxolone (100 mg/kg) (31.7 ± 2.3 µm^2^) and (-)-Carveol (100 mg/kg) (31, 7 ± 5.5 µm^2^) compared to the control group (Figure 4B). In the assessment of basic mucins in the gastric mucosa, a significant reduction was observed in the control group (5% tween 80) to 32.4 ± 6.4 µm^2^ compared to the normal group (87.2 ± 8.9 µm^2^). However, carbenoxolone (100 mg/kg) and (-)-Carveol (100 mg/kg) significantly increased mucins in the gastric mucosa region to 79.1 ± 7.0 µm^2^ and 79.2 ± 4.6 µm^2^, respectively, when compared to the control group (Figure 4C).

### NSAID and Stress-Induced Gastric Ulcer

Results obtained from piroxicam-induced gastric ulcer model demonstrated that oral administration of standard drug ranitidine (50 mg/kg) and (-)-Carveol (25, 50, 100 and 200 mg/kg) showed a significant reduction (*p* < 0.001) of the ULI in 52% (56.8 ± 7.3), 14% (101.4 ± 5.9), 30% (83.0 ± 4.3), 59% (48.6 ± 2.9) e 60% (47.2 ± 3.2), respectively, compared to the control group (5% tween 80) (118.6 ± 5.2) (Table 2). The same was observed in immobilization and cold-induced gastric ulcer model, in which oral administration of ranitidine (50 mg/kg) and (-)-Carveol (25, 50, 100 and 200 mg/kg) reduced the rate of ULI when compared to the control group in 52% (94.0 ± 9.5), 18% (159.7 ± 6.1), 43% (117.7 ± 7.0), 61% (76.7 ± 10.7) e 62% (76.0 ± 12.4), respectively (Table 2).

**TABLE 2:** Gastroprotective effect of different gastric lesion inducers in mouse.

Models	Treatments (p.o.)	Dose (mg/kg)	ULI	Inhibition (%)
NSAIDs (piroxicam)	5% Tween 80	—	118.6 ± 5.2	
	Ranitidine	50	56.8 ± 7.3***	52
	(-)-Carveol	25	101.4 ± 5.9***	14
		50	83.0 ± 4.3***	30
		100	48.6 ± 2.9***	59
		200	47.2 ± 3.2***	60
Stress (cold/restraint)	5% Tween 80	—	195.7 ± 10.6	
	Ranitidine	50	94.0 ± 9.5***	52
	(-)-Carveol	25	159.7 ± 6.1***	18
		50	111.7 ± 7.0***	43
		100	76.7 ± 10.7***	61
		200	76.0 ± 12.4***	62

Results are expressed as mean ± SD. One-way analysis of variance (ANOVA): followed by Dunnett’s and Tukey’s test was performed using the software Graph Pad Prism. ***p < 0.001 compared to the control group (5% Tween 80). (*n* = 8/per group). ULI, Ulcerative Lesion Index.

### Pyloric Ligature-Induced Gastric Ulcer

From the results obtained in this induction model it was possible to observe that ranitidine (50 mg/kg) and (-)-Carveol (100 mg/kg) administered orally reduced the ULI by 55 and 60%, respectively, in comparison to the 5% tween 80 group. When administered intraduodenally, ranitidine and (-)-Carveol reduced the ULI by 47 and 61%, respectively, compared to the control group (Table 3).

**TABLE 3:** Effect of oral and intraduodenal administration of ranitidine and (-)-Carveol in gastric ulcers induced by pyloric ligation in rats.

Treatment	Route of administration	Dose (mg/kg)	ULI	Inhibition (%)
5% Tween 80	Oral	—	366.6 ± 20.03	
Ranitidine	Oral	50	162.7 ± 24.1***	55
(-)-Carveol	Oral	100	143.8 ± 14.8***	60
5% Tween 80	Intraduodenal	—	340.2 ± 16.9	
Ranitidine	Intraduodenal	50	179.1 ± 25.3^###^	47
(-)-Carveol	Intraduodenal	100	132.4 ± 15.5^###^	61

The results are expressed as mean ± SD. One-way analysis of variance (ANOVA) followed by Dunnett’s test: ***p < 0.001 compared to the 5% tween 80 group orally and ^###^ *p* <0.001 compared to the 5% tween 80 group intraduodenal, (*n* = 8–10). ULI, Ulcerative Injury Index.

### Mechanisms of Action Involved in the Gastroprotective Activity of (-)-Carveol

#### Gastroprotective Activity of (-)-Carveol Involves a Modulation of Volume of Gastric Content in Pyloric Ligature-Induced Gastric Ulcer

From the pyloric ulcer induction protocol, the following biochemical parameters of the stomach contents of rats were determined: pH, [H+] and volume of gastric juice after administration of 5% tween 80, ranitidine (50 mg/kg) or (-)-Carveol (100 mg/kg) by oral and intraduodenal routes. With the results obtained in this protocol in both routes of administration (p.o. and i.d.), (-)-Carveol did not change the pH or [H+] when compared to the control group (5% Tween 80). However, it reduced the volume of gastric content compared to the 5% Tween 80 group. The groups treated with the ranitidine standard (50 mg/kg), regardless of the route of administration, showed an increase in pH, a decrease in [H+] and gastric volume when compared with their respective controls (Table 4).

**TABLE 4:** Effect of oral and intraduodenal administration of ranitidine and (-)-carveol on gastric content parameters after pyloric ligation in rats.

Treatment	Route of administration	Dose (mg/kg)	pH	[H^+^] (mEq/ml/4)	Volume (ml)
5% Tween 80	Oral	—	3.44 ± 0.29	8.43 ± 0.95	1.53 ± 0.11
Ranitidine	Oral	50	7.09 ± 0.43***	2.23 ± 0.93***	1.10 ± 0.07***
(-)-Carveol	Oral	100	3.21 ± 0.36	7.28 ± 0.89	1.17 ± 0.04***
5% Tween 80	Intraduodenal	—	2.83 ± 0.28	7.62 ± 0.87	1.33 ± 0.04
Ranitidine	Intraduodenal	50	5.03 ± 0.38^###^	3.59 ± 0.53^###^	0.74 ± 0.05^###^
(-)-Carveol	Intraduodenal	100	2.90 ± 3.23	7.70 ± 0.63	1.00 ± 0.02^###^

The results are expressed as mean ± SD. One-way analysis of variance (ANOVA) followed by Dunnett’s test: ***p < 0.001 compared with the 5% tween 80 group orally and ^###^ *p* < 0.001 compared with the 5% tween 80 group intraduodenal (*n* = 8–10).

#### Cytoprotective Mechanisms

##### Involvement of Sulfhydryl Groups

**FIGURE 5:**
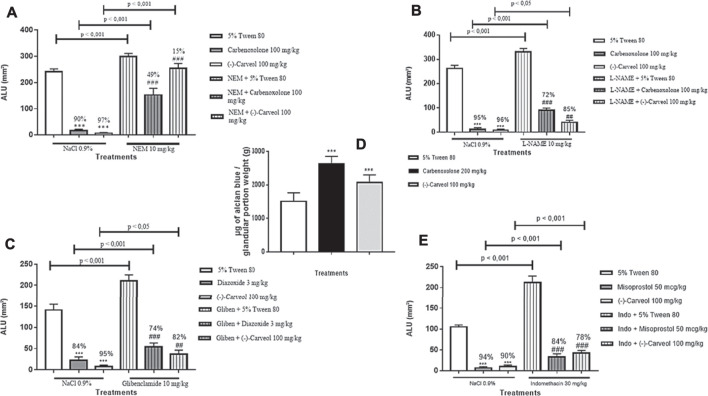
Influence of the oral pre-treatment with (-)-Carveol in rats on ulcer-associated molecular pathways. **A** – Sulfhydryl groups; **B** – Nitric oxide; **C** – ATP-dependent potassium channels; **D** – Secretion of mucus; **E** – Prostaglandins. The results are expressed as mean ± SD. One-way analysis of variance (ANOVA) followed by Dunnett's and Tukey's test was performed using the software Graph Pad Prism. ***p < 0.001, compared to the control group not blocked. ###p < 0.001, compared to the block + control group (n = 6-7/per group). The statistical difference between the unblocked and blocked groups is expressed in the horizontal bars above each group and was verified by the Tukey's test. The percentage of inhibition was calculated in relation to the respective control group.

Groups previously treated with the carbenoxolone (100 mg/kg) and (-)-Carveol (100 mg/kg) showed a significant reduction (*p* < 0.001) in ULA by 90% (17.2 ± 4.6 mm^2^) and 97% (6.8 ± 2.3 mm^2^), respectively, when compared to the control group (5% tween 80) (242.3 ± 9.7 mm^2^). However, when NEM (10 mg/kg), an inhibitor of the sulfhydryl groups, was administered, there was an exacerbation of ULA (299.7 ± 11.5 mm^2^) with a reduction in the gastroprotective effect of carbenoxolone (100 mg/kg) and (-)-Carveol (100 mg/kg) for 49% (152.5 ± 25.1 mm^2^) and 15% (254.7 ± 18.2 mm^2^), respectively, compared to the groups in which the blocker was not administered (Figure 5A).

##### Involvement of Nitric Oxide

Carbenoxolone (100 mg/kg) and (-)-Carveol (100 mg/kg) showed a significant reduction (*p* < 0.001) in ULA by 95% (11.4 ± 7.5 mm^2^) and 96% (9,1 ± 3.0 mm^2^), respectively, when compared to the control group (5% tween 80) (263.0 ± 12.5 mm^2^). However, previous administration of L-NAME, a blocker of NO synthesis, reduced the gastroprotection exerted by carbenoxolone and (-)-Carveol to 72% (90.8 ± 8.3 mm^2^) and 85% (40, 9 ± 7.7 mm^2^), respectively, when compared to groups that did not receive the blocker (Figure 5B).

##### Involvement of the ATP-Dependent Potassium Channels

(-)-Carveol (100 mg/kg) and diazoxide (3 mg/kg), an agonist of the KATP channels, significantly reduced (*p* < 0.001) the area of ethanol-induced ulcerative injury by 95% (7.5 ± 2.6 mm^2^) and 84% (22.6 ± 7.9 mm^2^), respectively, when compared to the negative control group (tween 80 5%) (140.9 ± 14.0 mm^2^). Glibenclamide, an antagonist of the KATP channels, reduced the gastroprotective effect of (-)-Carveol and diazoxide to 82% (37.3 ± 8.9 mm^2^) and 74% (53.9 ± 9.6 mm^2^), respectively, both compared to the non-blocked groups (Figure 5C).

##### Mucus Secretion Participates in the Gastroprotection Exerted by (-)-Carveol

The mucus adhered to the gastric mucosa was indirectly quantified by the amount of alcian blue in the stomach gland. In this model, carbenoxolone (200 mg/kg) and (-)-Carveol (100 mg/kg) increased the mucus adhered to the stomach wall by 2,621 ± 230 and 2074 ± 227 μg of alcian blue/weight of the glandular portion (g), respectively, when compared to the control group (5% tween 80) (1,510 ± 256 μg of alcian blue/weight of the glandular portion) (Figure 5D).

##### Involvement of Prostaglandins

Misoprostol (50 μg/kg) and (-)-Carveol (100 mg/kg) reduced ULA by 94% (6.5 ± 3.2 mm^2^) and 90% (10.3 ± 3.5 mm^2^), respectively, when compared to the control group (5% tween 80) (105.1 ± 4.7 mm^2^). However, when the animals were submitted to treatment with indomethacin (30 mg/kg), a non-selective cyclooxygenase blocker (COX), there was an exacerbation of ULA (211.9 ± 15.9 mm^2^) with reduced gastroprotection of misoprostol and (-)-Carveol to 84% (32.6 ± 8.0 mm^2^) and 78% (42.7 ± 5.9 mm^2^), respectively, when com-pared to the non-blocked groups (Figure 5E).

#### Antioxidant Mechanisms Involved in the Gastroprotective Activity of (-)-Carveol

##### Reduced Glutathione

**FIGURE 6:**
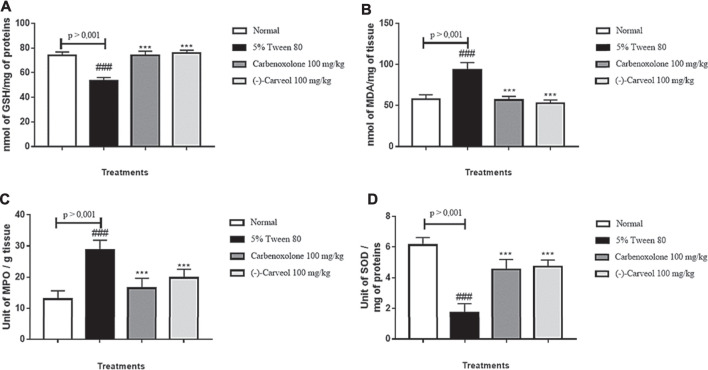
Effect of oral administration of carbenoxolone and (-)-carveol on antioxidants levels from the ethanol-induced gastric ulcer model in rats. **A** – GSH; **B** – MDA; **C** – MPO; **D** – SOD. Results are expressed as mean ± s.p.m. One-way analysis of variance (ANOVA) followed by Dunnett's and Tukey's test was performed using the software Graph Pad Prism. ***p < 0.001 compared to the control group (5 % tween 80); ###p < 0.001 compared to the normal group. (n = 6/per group).

In determining the antioxidant activity of (-)-Carveol, it was possible to verify that the group of animals pre-treated with the vehicle alone (5% tween 80) showed a reduction in GSH levels to 53.5 ± 2.5 nmol of GSH/mg protein compared to the normal group (73.8 ± 2.9 nmol GSH/mg protein). However, previous administration of carbenoxolone (100 mg/kg) (74.0 ± 3.3 nmol of GSH/mg of proteins) and (-)-Carveol (100 mg/kg) (75.6 ± 2.5 nmol GSH/mg protein) restored baseline GSH levels (Figure 6A).

##### Malondialdehyde

The group 5% tween 80 showed an increase in MDA levels to 93.4 ± 8.7 nmol MDA/g tissue compared to the normal group (57.6 ± 5.4 nmol MDA/g tissue). However, the administration of carbenoxolone (100 mg/kg) (56.5 ± 4.6 nmol of MDA/g tissue) and (-)-Carveol (100 mg/kg) (52.7 ± 3.9 nmol MDA/g of tissue) restored MDA levels to baseline conditions (Figure 6B).

##### Myeloperoxidase

The results showed that in the control group (5% tween 80) there was an increase in MPO levels to 28.7 ± 3.1 units of MPO/g of tissue when compared to the normal group (12.9 ± 2.7 units MPO/g tissue). However, the groups treated with carbenoxolone (100 mg/kg) and (-)-Carveol (100 mg/kg) significantly reduced MPO levels to 16.4 ± 3.2 and 19.7 ± 2.7 unit of MPO/g of tissue, respectively, in relation to the control group (Figure 6C).

##### Superoxide Dismutase

Animals in the control group (5% tween 80) demonstrated a significant reduction in the activity of the SOD enzyme to 1.72 ± 0.55 U of SOD/mg of protein compared to the normal group (6.12 ± 0.49 U SOD/mg protein). However, SOD activity was significantly increased (*p* < 0.001) with previous administration of carbenoxolone (100 mg/kg) and (-)-Carveol (100 mg/kg) to 4.52 ± 0.65 and 4, 72 ± 0.41 U of SOD/mg of protein, respectively, when compared to the control (5% tween 80) (Figure 6D).

#### Immunoregulatory Mechanisms Involved in the Gastroprotective Activity of (-)-Carveol

##### Levels of IL-1β, TNF-α and IL-10

**FIGURE 7:**
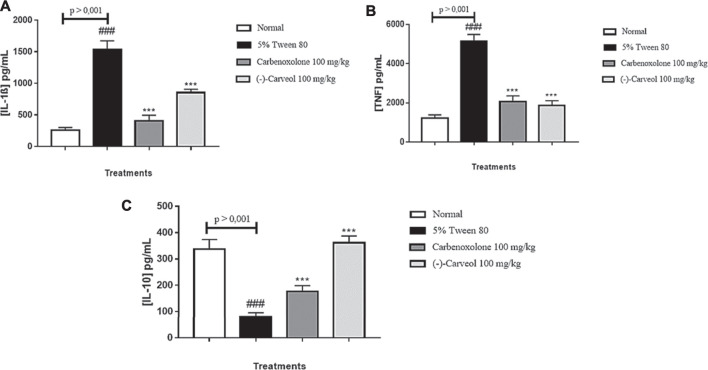
Effect of oral administration of carbenoxolone and (-)-carveol on the levels of IL-1β (A), TNF-α (B) and IL-10 (C) from the gastric ulcer model induced by ethanol in rats. Results are expressed as mean ± s.d. One-way analysis of variance (ANOVA) followed by the Dunnett and Tukey's test was performed using the software Graph Pad Prism. ***p < 0.001 compared to the control group (5 % tween 80); ###p < 0.001 compared to the normal group; (n = 6/per group).

The control group (5% tween 80) increased the levels of the pro-inflammatory interleukin IL-1β to 1538.1 ± 136.7 pg/ml compared to the normal group (257.8 ± 42.2 pg/ml). Administration of carbenoxolone (100 mg/kg) and (-)-Carveol (100 mg/kg) reduced (*p* < 0.001) IL-1β levels to 407.8 ± 88.5 and 853.0 ± 52.9 pg/ml, respectively, when compared to the 5% tween 80 group (Figure 7A). A similar result was found for TNF-α, in which the control group (5% tween 80) showed a significant increase (5142.1 ± 347.3 pg/ml) in TNF-α levels when compared to the normal group (1,230.2 ± 164.7 pg/ml). Treatments with carbenoxolone and (-)-Carveol reduced (*p* < 0.001) TNF-α levels to 2074.1 ± 283.0 and 1860.1 ± 253.0 pg/ml respectively, when compared to the treated group only with 5% tween 80 (Figure 7B). Regarding IL-10 levels, a significant reduction was observed in the control group (5% tween 80) (79.2 ± 16.7 pg/ml) compared to the normal group (337.7 ± 36.4 pg/ml). Treatment with carbenoxolone promoted a significant increase (176.3 ± 22.6 pg/ml) in IL-10 levels compared to the group treated with 5% tween 80. The administration of (-)-Carveol reestablished baseline levels of IL-10 (361.9 ± 25.4 pg/ml) (Figure 7C).

## Discussion

Acute toxicity tests on animals show unwanted effects immediately or in a short time after a single or multiple administration of the substance within 24 h by a specific route. The unwanted effect includes any effect that leads to behavioral, nutritional (reduction in water and feed consumption), on organs and/or biochemistry changes. Signs of systemic toxicity also involve reduced body mass, apathy and poor coat condition, such as the presence of piloerection (Rajeh et al., 2012). Another toxicity parameter evaluated in non-clinical animal tests is the estimated 50% lethal dose (LD50), characterized as the amount of a substance capable of causing the death of 50% of the animals under experiment, during a specified period of time (Hayes and Kriger, 2014; Adamson, 2016).

The results suggest that (-)-Carveol is safe to use when administered under the evaluated conditions. The approximate LD50 for (-)-Carveol is equal to or greater than 2,500 mg/kg, which makes it fall into category 5, according to the Global Harmonized Classification System (GHS). According to Kennedy et al., a substance that has an LD50 greater than 2000 mg/kg orally can be considered of low toxicity (Kennedy et al., 1986).

Moreover, literature data on acute oral toxicity in rats estimate an LD50 of 3,000 mg/kg for (-)-Carveol (Bhatia et al., 2008). In the scientific literature, numerous studies have already demonstrated the low toxicity of terpenes (Belsito et al., 2008), as the monoterpene limonene, which was considered a low toxicity chemical based on LD50 and in repeated dose toxicity studies when administered orally to animals (Kim et al., 2013).

Once the acute oral toxicity was determined, the gastroprotective activity of (-)-Carveol was evaluated against different experimental models of gastric lesions. The development of linear haemorrhagic lesions, extensive swelling in the submucosal layer, infiltration of inflammatory cells and loss of gastric epithelial cells are typical features of ethanol-induced damage. This tissue injury promoted by ethanol occurs through different mediators, such as cytokines and reactive oxygen species (ROS), decreased mucus, and depletion of sulfhydryl groups (Abdelfattah et al., 2019). ROS stimulated in the presence of ethanol are responsible for lipid peroxidation and chemotaxis of inflammatory cells to the injury site. Ethanol induces leukocyte recruitment that stimulates inflammatory responses by increasing levels of pro-inflammatory cytokines such as TNF-α and IL-1β (Song et al., 2019).

By the data obtained thought this model is possible to suggest that (-)-Carveol plays a protective role in the gastric mucosa. Cytoprotective, anti-secretory, antioxidant and/or immunoregulatory mechanisms are possibly associated with the antiulcer property demonstrated here. Similar data around gastroprotective activity in the ethanol model were observed with levogyrous monoterpenes (-)-linalool (da Silva et al., 2016) and (-)-myrtenol (Viana et al., 2016). Besides, the monoterpenes carvacrol (Oliveira et al., 2012), menthol (Rozza et al., 2013), geraniol (de Carvalho et al., 2014), thymol (Chauhan and Kang, 2015), 1,8-cineole (Rocha Caldas et al., 2015), α-pinene (Pinheiro et al., 2015), citral (Venzon et al., 2018) and limonene (de Souza et al., 2019) have shown a reduction in gastric lesions induced by ethanol.

Histomorphological analysis showed that the administration of (-)-Carveol reduced all inflammatory parameters evaluated and increased mucus production by PAS staining, which acts to protect the stomach against aggressive agents such as ethanol, suggesting a cytoprotective effect.

The gastric lesion induced by ethanol promotes an inflammatory process involving mast cells, which after being activated release substances such as histamine, leukotrienes and platelet activating factor. When histamine is released by mast cells in the submucosal layer, it stimulates an additional production of gastric acid when interacting with H2 receptors (Ahmed et al., 2020). Other studies that evaluated the role of mast cells in gastric lesions also revealed an increase in this type of cells caused by the administration of ethanol. This in-crease is probably related to the severity of the inflammation and ulcer (Ribeiro et al., 2016; Yang et al., 2017). The reduction in the number of mast cells in the stomach segment after treatment with (-)-Carveol observed in staining with toluidine blue confirms its potential contribution in reducing the inflammatory response.

Intragastric administration of ethanol leads to destabilization of gastric vascular homeostasis due to increased endothelial activity, as well as disorganization and deposition of the extracellular matrix. These events lead to blood leakage and infiltration of inflammatory cells. This infiltration of inflammatory cells from the circulation into the tis-sues due to impaired vascular homeostasis also contributes to the development of gastric ulcers (Yao et al., 2019). In that study, the administration of ethanol led to severe bleeding and an increase in the number of mast cells in the entire submucosal layer that is swollen. To determine whether the occurrence of haemorrhage and edema was due to the imbalance of vascular homeostasis, we sought to evaluate the deposition of the extracellular matrix using Masson’s Trichrome stain. The data obtained reveal that in the control group (5% tween 80) there was an increase in deposition of extracellular matrix. However, treatment with (-)-Carveol reduced this deposition. These results indicate that this phytoconstituent plays an important role in regulating the deposition of the extracellular matrix and, possibly, in the regulation of gastric vascular homeostasis.

Similar to ethanol, the stress ulceration mechanism is complex, multifactorial and involves factors such as individual vulnerability, stimulation of specific brain pathways that regulate autonomic function, increased muscle contractility, reduced blood flow to the mucosa, mast cell degranulation, leukocyte activation, ROS generation and lipid peroxidation (Elshazly et al., 2018). Of these mechanisms, insufficient microcirculation in the gastric mucosa is considered the main cause of reduced mucosal defence mechanisms and ulcer formation. In addition, reactive oxygen species (ROS) increase in ischemic tissue and are mediators of stress-induced gastrointestinal injuries. ROS trigger lipid peroxidation with subsequent loss of membrane integrity and cell death (Lin et al., 2020). (-)-Carveol showed a gastroprotective effect in all doses evaluated, which suggests that gastric cytoprotection may be involved in this effect. Similar results were seen in the studies with the laevorotatory monoterpene (-)-myrtenol (Viana et al., 2016).

Gastric ulcers resulting from the continuous use of NSAIDs may be related to mechanisms that include a local damaging action for destabilizing the membrane phospho-lipid layer and damaging the mucus barrier, favouring the retro-diffusion of H^+^ ions that damage the surface cells of the gastric mucosa, inhibition of the enzyme cyclo-oxygenase (COX) and consequently the reduction of the synthesis of cytoprotective prostaglandins (PGE2 and PGI2), which act by stimulating the production of mucus and bicarbonate, in maintaining blood flow and in the negative regulation of gastric acid secretion (Bjarnason et al., 2018). (-)-Carveol reduced gastric injuries induced by piroxicam, suggesting that this monoterpene may act by a cytoprotective mechanism. These results corroborate some reports in the literature, in which, the monoterpenes carvacrol (Oliveira et al., 2012), 1,8-cineole (Rocha Caldas et al., 2015), thymol (Chauhan and Kang, 2015; Ribeiro et al., 2016; Geyikoglu et al., 2018; Koc et al., 2020) and menthol (Rozza et al., 2013; Rozza et al., 2014) showed gastroprotective activity in this induction model.

Gastric acid secretion is a key element in the formation of ulcers in the pyloric ligation model. One of the mechanisms associated with this effect is the reflex vagal stimulation through the pressure receptors located in the antral gastric mucosa, causing the release of the secretagogue acetylcholine. As well, gastric distension produced by the accumulation of secretion seems to influence the gastric acid secretion of this model, possibly by increasing the release of gastrin hormone, a potent stimulator of parietal cells to produce hydrochloric acid. Pyloric ligation leads to the accumulation of gastric acid and pepsin, favouring self-digestion of the gastric mucosa and ulceration (Rajakrishnan et al., 2020).

(-)-Carveol reduced the ulcerative lesion when administered orally and intraduodenally, suggesting that this phytoconstituent exerts its gastroprotective effect by both local and systemic action. These results corroborate the study conducted with the essential oil of *Croton cajucara*, consisting of phenylpropanoid or terpenoid derivatives, mainly monoterpenes and sesquiterpenes, which reduced gastric injury in this model (Nascimento et al., 2017). Treatment with (-)-Carveol did not alter the pH and the concentration of H+ ions, however, it reduced the volume of gastric juice in both routes. Thus, it can be inferred that gastroprotection promoted by (-)-Carveol is associated with local and systemic antisecretory mechanisms. These data suggest that (-)-Carveol is a potential antiulcer agent because it reduces gastric acid secretion without modulating the pH and concentration of H+ ions, which are important parameters for several physiological processes such as protein activation (pepsin) and absorption of essential nutrients (vitamin B12). Monoterpene 1,8-cineole showed similar result (Rocha Caldas et al., 2015).

In the evaluation of the cytoprotective mechanisms involved in the gastroprotective activity of (-)-Carveol, the role of the sulfhydryl groups, nitric oxide, KATP, mucus and prostaglandins were investigated.

The sulfhydryl groups (SHs) continuously adhere to the mucus layer, forming a kind of stable protective barrier against the action of pepsin and luminal hydrochloric acid, preventing proteolytic digestion of the underlying mucosa. With the reduction of SHs linked to mucin, the mucus becomes soluble and easily removed by harmful agents (Liang et al., 2018). The results obtained suggest that sulfhydryl groups are key mediators in the defence of gastric mucosa induced by (-)-Carveol, since pre-treatment with NEM, an inhibitor of sulfhydryl groups has reversed its gastroprotective effect. The monoterpenes menthol (Rozza et al., 2014), geraniol (de Carvalho et al., 2014) and 1,8-cineole (eucalyptol) (Rocha Caldas et al., 2015) also demonstrated gastroprotective effect related to SHs.

Nitric oxide (NO) is already well described as a crucial factor in the defence of the gastrointestinal mucosa. NO-mediated gastroprotection involves inhibiting neutrophil adhesion, stimulating gastric mucus secretion and increasing blood flow in the mucosa. Studies report that donors of NO and L-arginine exert a gastroprotective effect, accelerating the healing process of ulcers, while inhibition of NO by L-NAME in experimental protocols aggravate the injury (Silva et al., 2014). The results obtained suggest that NO participates, at least in part, in the gastroprotective effect of (-)-Carveol since, in the presence of the inhibitor L-NAME, its effect was partially reduced. Other monoterpenes also exhibited this behaviour such as carvacrol (Oliveira et al., 2012), geraniol (de Carvalho et al., 2014) and (-)-myrtenol (Viana et al., 2016).

The opening of the KATP channels participates in the protection of the gastric mucosa by relaxing the vessels that supply the mucosa, leading to an increase in gastric blood flow. This effect is suppressed by the administration of glibenclamide, a KATP channel blocker (Arunachalam et al., 2019). The animals pre-treated with (-)-Carveol and glibenclamide, had their protective effects reduced, suggesting that the KATP channels are partially involved in the gastroprotective activity of this monoterpene. These data are similar to the results obtained with carvacrol (Oliveira et al., 2012), menthol (Rozza et al., 2013), and thymol (Ribeiro et al., 2016).

The surface of the gastric mucosa is covered by a layer formed of mucus, bicarbonate and phospholipids constituting the first line of defense of the gastric mucosa (Malfertheiner and Schulz, 2020). The mucus protects the gastric mucosa both for its viscosity, protecting against irritating substances, the interaction with invading pathogens or mechanical injuries, and for its alkaline character, preventing the proteolytic action of pepsin (Barrett, 2018). (-)-Carveol increased the amount of gastric mucus, as evidenced by the measurement of mucus molecules adhering to alcian blue in the gastric mucosa and also by the histomorphological analysis of slides stained with PAS, suggesting that the gastroprotective effect of this monoterpene involves the stimulation of this agent cytoprotective. These results corroborate the findings of the monoterpenes carvacrol (Oliveira et al., 2012), menthol (Rozza et al., 2013; Rozza et al., 2014), geraniol (de Carvalho et al., 2014), thymol (Ribeiro et al., 2016), and 1,8-cineole (Rocha Caldas et al., 2015).

Prostaglandins, especially PGE2 and PGI2, are produced continuously via COX by the gastric epithelium and participate in the maintenance of stomach homeostasis and the healing of ulcers by acting by inhibiting gastric acid secretion and increasing mucus and bicarbonate production (Koji and Kikuko, 2018). Indomethacin exacerbated the ulcerative lesion and moderately reduced the effects of (-)-Carveol. Similar results were found with the monoterpenes carvacrol [53], menthol (Rozza et al., 2013; Rozza et al., 2014), geraniol (de Carvalho et al., 2014), (-)-myrtenol (Viana et al., 2016), 1,8-cineole (Rocha Caldas et al., 2015) and thymol (Chauhan and Kang, 2015; Ribeiro et al., 2016; Geyikoglu et al., 2018; Koc et al., 2020), suggesting that the gastroprotective property shown by these monoterpenes involves the modulation of prostaglandins.

Together, these data indicate that (-)-Carveol has gastroprotective activity related to cytoprotective properties, mainly involving a reinforcement of SHs in the gastric mucosa and increased mucus secretion and involves, at least partially, NO, KATP and prostaglandins.

In order to characterize the probable antioxidant effect of (-)-Carveol against gastric damage, markers of oxidative stress such as GSH, MDA, SOD and MPO were evaluated. Oxidative stress is generated by the imbalance between the production and neutralization of pro-oxidant and antioxidant agents, being associated with an increase in free radicals and reactive oxygen species (ROS). Excessive production of ROS in gastric tissue disturbs the integrity and permeability of biomembranes, leading to cell death and favoring the formation of ulcers (Wang et al., 2018). The cells of the gastric mucosa have an antioxidant defence system that aims to prevent or minimize the cytotoxicity promoted by oxidative stress. This system involves the action of enzymes such as SOD and compounds with the potential to scavenge free radicals such as GSH (do Carmo et al., 2018).

GSH is considered the first non-enzymatic line of defence of the organism against ROS (Liang et al., 2018). An increase in the concentration of GSH prevents damage caused by ROS, while its reduction is associated with a great vulnerability of cells to ROS (Kountouras et al., 2017). In this work, (-)-Carveol increased the levels of GSH in gastric tissue samples. Menthol (Rozza et al., 2013; Rozza et al., 2014) and geraniol (de Carvalho et al., 2014) also increased levels of GSH.

SOD is one of the most important antioxidant enzymes. This enzyme exerts its effect through the dismutation of the superoxide radical anion, generated by cellular metabolism or by external sources, transforming it into molecular oxygen and hydrogen peroxide, which is less reactive and can be degraded by other antioxidant system enzymes like catalase (Liang et al., 2018). (-)-Carveol plays a protective role by increasing SOD activity and consequently potentiating the dismutation of the superoxide anion. The increase in SOD activity, makes (-)-Carveol a gastroprotective substance with an effect on the antioxidant system, constituting one of the mechanisms of action of this compound. There are many reports of the importance of natural compounds with antioxidant properties to reduce complications caused by oxidative stress in gastric tissues such as (-)-myrtenol (Viana et al., 2016), α-boswélic acid (Zhang et al., 2016), (-)-α-bisabolol (Rocha et al., 2011) and patchoulene epoxide (Liang et al., 2018) which increased SOD levels.

MDA is one of the most common markers of lipid peroxidation and oxidative stress that can be quantified in ulcerated and inflammatory lesions of the gastrointestinal tract (Song et al., 2019). The results obtained showed that the lipid peroxidation induced by ethanol was reduced by pre-treatment with (-)-Carveol. Similar to monoterpenes 1,8-cineole (Rocha Caldas et al., 2015) and (-)-myrtenol (Viana et al., 2016).

MPO is expressed mainly in neutrophils and to a lesser extent in monocytes. One of the main effects of MPO and its final product, hypochlorous acid (HOCl), is the formation of free radicals, such as chloramines that are involved in oxidative damage and pro-inflammatory responses (Aratani, 2018). (-)-Carveol showed a reduction in MPO levels, an indirect marker of neutrophil infiltration. Similar to monoterpenes limonene (de Souza et al., 2019), menthol (Rozza et al., 2013; Rozza et al., 2014) and geraniol (de Carvalho et al., 2014).

TNF-α plays an important role in the development of ulcerative lesions when initiating an acute inflammatory response, accompanied by neutrophilic infiltration in the gastric mucosa (Mantovani et al., 2019). In addition, it modulates the apoptosis process in the gastric mucosa by activating the caspase-3 pathway, suppressing gastric microcirculation, cell proliferation and angiogenesis at the ulcer margin, delaying the healing of the ulcer. The exacerbated expression of IL-1β contributes to the formation of ulcers and stimulates the production of more TNF-α (Boshtam et al., 2017). IL-10 protects the gastric mucosa, by signalling an anti-inflammatory response, reducing the immune response mediated by pro-inflammatory cells. This effect occurs through the inhibition of TNF-α feedback and the consequent re-duction of the inflammatory process in gastric tissue (Mollazadeh et al., 2019).

Treatment with (-)-Carveol reduced the levels of pro-inflammatory cytokines (TNF-α and IL-1β) and increased the regulatory cytokine IL-10 back to baseline levels, suggesting an interesting immunomodulatory effect. These findings may also be related to a reduction in the infiltration of inflammatory cells, such as neutrophils, reflected by the decrease in MPO activity in the group treated with (-)-Carveol in response to gastric damage induced by ethanol. The monoterpenes limonene (de Souza et al., 2019) and thymol (Chauhan and Kang, 2015) they also exhibited immunoregulatory activities by decreasing the levels of TNF-α and IL-1β and increasing the level of IL-10.

(-)-Carveol, a substance originally derived from natural products, has an important gastroprotective activity involving multiple mechanisms of action, such as cytoprotective mechanisms (participation of sulfhydryl groups, nitric oxide, KATP channels, mucus secretion and prostaglandins), antisecretory (reduction in the volume of gastric secretion), antioxidants (increase in GSH and SOD with reduction in MDA and MPO) and immunoregulatory (reduction in TNF-α and IL-1β and increase in IL-10).

## Data Availability

The original contributions presented in the study are included in the article/Supplementary Material, further inquiries can be directed to the corresponding author.

## Abbreviations

COX-2, cyclooxygenase 2; GSH, non-protein sulfhydryl group; HE, hematoxylin and eosin; HTAB, trimethylammonium bromide hexadecyl buffer; id, intraduodenal; iv, intravenous; IL-10, interleukin10; IL-1β, interleukin1beta; KATP, ATP-dependent potassium channels; LD50, lethal dose; L-NAME, N-nitro-L-arginine methyl ester; MDA, malondialdehyde; MPO, myeloperoxidase; NEM, N-ethylmaleimide; NF-kB, nuclear transcription factor kappa B; NO, nitric oxide; NSAID, non-steroidal anti-inflammatory; OECD, organization for co-operation and development; po, oral; PAS, Schiff’s periodic acid; pH, hydrogen potential; SHs, sulfhydryl groups; SOD, superoxide dismutase; TNF-α, tumor necrosis factor alpha; ULA, ulcerative lesion area; ULI, ulcerative lesion index.
